# Anti-Parasitic Compounds from *Streptomyces* sp. Strains Isolated from Mediterranean Sponges

**DOI:** 10.3390/md8020373

**Published:** 2010-02-23

**Authors:** Sheila Marie Pimentel-Elardo, Svitlana Kozytska, Tim S. Bugni, Chris M. Ireland, Heidrun Moll, Ute Hentschel

**Affiliations:** 1 Julius-von-Sachs Institute for Biological Sciences, University of Würzburg, Julius-von-Sachs Platz 3, 97082 Würzburg, Germany; 2 Research Center for Infectious Diseases, Josef-Schneider-Straße 2, 97080 Würzburg, Germany; 3 Department of Medicinal Chemistry, University of Utah, Salt Lake City, 84112 Utah, USA

**Keywords:** marine sponges, Streptomyces, valinomycin, staurosporine, butenolide, anti-parasitic

## Abstract

Actinomycetes are prolific producers of pharmacologically important compounds accounting for about 70% of the naturally derived antibiotics that are currently in clinical use. In this study, we report on the isolation of *Streptomyces* sp. strains from Mediterranean sponges, on their secondary metabolite production and on their screening for anti-infective activities. Bioassay-guided isolation and purification yielded three previously known compounds namely, cyclic depsipeptide valinomycin, indolocarbazole alkaloid staurosporine and butenolide. This is the first report of the isolation of valinomycin from a marine source. These compounds exhibited novel anti-parasitic activities specifically against *Leishmania major* (valinomycin IC_50_ < 0.11 μM; staurosporine IC_50_ 5.30 μM) and *Trypanosoma brucei brucei* (valinomycin IC_50_ 0.0032 μM; staurosporine IC_50_ 0.022 μM; butenolide IC_50_ 31.77 μM). These results underscore the potential of marine actinomycetes to produce bioactive compounds as well as the re-evaluation of previously known compounds for novel anti-infective activities.

## 1. Introduction

The class *Actinobacteria*, specifically bacteria belonging to the order *Actinomycetales*, are common soil inhabitants that have the unprecedented ability to produce a wide range of secondary metabolites. Among the more than 140 described actinomycete genera, only a few are responsible for the majority of over 20,000 microbial natural products identified so far. In particular, the genus *Streptomyces* accounts for about 80% of the actinomycete natural products reported to date [1,2]. Given the unparalleled potential of actinomycetes and specifically streptomycetes in this regard, significant effort has been directed towards the isolation of these bacteria from various sources for drug screening programs. The majority of the actinomycetes were previously isolated from terrestrial soils and from marine sediments [3–5] and quite recently also from marine sponges [6–10] and cone snails [11]. The discovery of numerous marine actinomycete taxa and their bioactive secondary metabolites dispel the notion that actinomycetes are merely dormant spores that have been washed off from the shores [1,3,12,13].

The major goal of our research is to discover novel anti-infective agents such as those against the parasites *Leishmania major* and *Trypanosoma brucei* that cause leishmaniasis and African sleeping sickness, respectively. These parasites currently affect around 12 million people living in tropical and subtropical areas [14]. The alarming death rate caused by these parasites and the emergence of antibiotic resistance underline the need for new and effective drugs. Our research program focuses on the discovery of anti-infective agents from marine sponges and their associated microorganisms. In the course of our study, we have taxonomically described two new actinomycete species [15,16] isolated from marine sponges as well as novel compounds [17]. During our screening efforts for bioactive natural products from marine sponge-associated actinomycetes, we have encountered some previously known compounds but with yet unprecedented biological activities. We report here the isolation and characterization of these compounds from actinomycetes associated with Mediterranean sponges with novel anti-parasitic activities.

## 2. Results and Discussion

**Figure 1:**
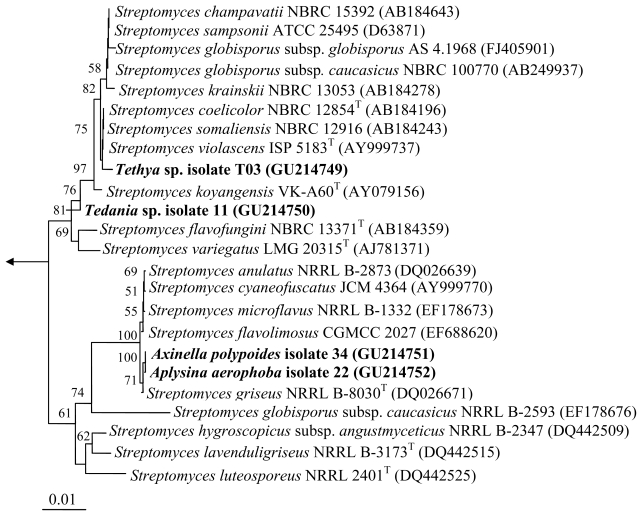
Neighbor-joining tree of the strains and representative species of the genus *Streptomyces* based on nearly complete 16S rRNA gene sequences. Numbers at the nodes indicate the levels of bootstrap support based on 1000 resampled data sets. Only values greater than 50% are shown. The arrow points to the outgroup consisting of six species belonging to *Enterobacteriaceae* and *Pasteurellaceae.* The scale bar indicates 0.01 substitution per nucleotide position.

Actinomycetes associated with the following sponges: *Aplysina aerophoba, Axinella polypoides, Tedania* sp. and *Tethya* sp. collected by SCUBA diving offshore Rovinj, Croatia (45°05′N, 13°38′E) in May 2006 were cultivated as described previously [15,16,18]. 16S rRNA gene sequencing revealed the affiliation of four strains, namely isolate 11 (GU214750), isolate 34 (GU214751), isolate 22 (GU214752) and isolate TO3 (GU214749) to the genus *Streptomyces* (Figure 1). They exhibited 99.7–99.9% sequence similarities to validly described species of the genus *Streptomyces*.

The strains 22 and 34 collected from two different sponge species, *Axinella polypoides* and *Aplysina aerophoba*, respectively exhibited 99.9% 16S rRNA gene sequence similarities, with only one nucleotide difference. This suggests that these isolates are most probably the same strain and their isolation from different hosts indicates that these bacteria could be transient organisms coming from the surrounding seawater that were merely present within the sponge during collection.

The strains 11, 22, 34 and T03 were each grown on 100 M1 [4] agar plates and incubated at 30 °C for seven days. Mycelial mass together with the agar were cut into small pieces and macerated overnight with 200 mL of ethyl acetate. The resulting solution was filtered using Whatman filter paper and the same maceration step with ethyl acetate was repeated. Both filtrates were combined and subsequently dried by rotary evaporation. The crude extracts were subjected to pre-fractionation with Diaion HP-20ss resin (Mitsubishi Chemical Corporation, Japan) using a gradient of water/isopropanol (100%, 75%:25%, 50%:50%, 25%:75%) followed by 100% MeOH. The fractions were subsequently purified by RP-HPLC (Agilent 1100, Agilent Technologies, USA). High resolution ESIMS analyses were performed on a Micromass Q-Tof micro mass spectrometer. NMR spectra were obtained on Varian INOVA 500 (^1^H 500 MHz, ^13^C 125 MHz) and Varian INOVA 600 (^1^H 600 MHz, ^13^C 150 MHz) NMR spectrometers with a 3 mm Nalorac MDBG probe and a 5 mm cold probe, respectively.

**Figure 2:**
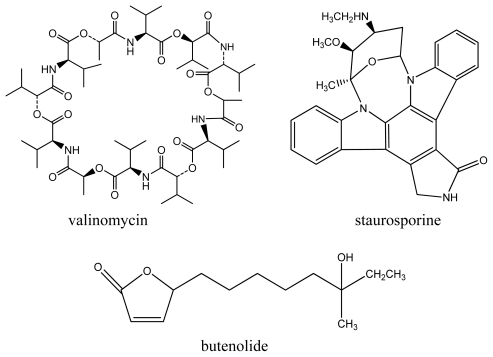
Compounds isolated from *Streptomyces* sp. strains.

High-resolution mass spectrometry of the purified compound both from *Streptomyces* sp. strains 22 and 34 established a molecular formula of C_54_H_90_N_6_NaO_18_ (*m/z* 1133.6385 for [M + Na]^+^, calculated 1133.6394) [19]. A combination of NMR and MS-MS fragmentation suggested the presence of one α-hydroxyisovaleryl unit (Hiv), a lactoyl group (Lac), and two valines (Val), thus confirming the identity of the compound as valinomycin (Figure 2) (see Supplementary Information for NMR data). The isolation of the same compound from these strains is not surprising since both exhibited very high 16S rRNA gene sequence similarities. This cyclodepsipeptide has been recovered from various soil-derived actinomycetes, *Streptomyces fulvissimus*, *Streptomyces roseochromogenes* and *Streptomyces griseus* var. *flexipartum* [20]. To date, this is the first report of valinomycin isolated from a marine organism. This cyclic depsipeptide consists of polar groups oriented toward the central cavity, whereas the rest of the molecule is relatively nonpolar thus behaving like an ionophore that modulates transport of ions such as potassium across biological membranes. In this study, valinomycin exhibited significant inhibitory activities against the parasites *Leishmania major* (IC_50_ < 0.11 μM) and *Trypanosoma brucei brucei* (IC_50_ 0.0032 μM) [21,22]. Previous studies have shown other biological activities of valinomycin in insecticidal, nematocidal and antifungal assays [23].

The compound staurosporine (Figure 2) was isolated from *Streptomyces* sp. strain 11 with a molecular formula of C_11_H_18_N_2_NaO_2_ (*m/z* 233.1262 for [M + Na]^+^, calculated 233.1266) [24]. The structure was confirmed by comparison of NMR analysis (see Supplementary Information) with published spectral data of the compound [25]. This indolocarbazole alkaloid was previously isolated from various terrestrial *Streptomyces* sp. strains. Interestingly, staurosporine and its derivatives have also been isolated from the marine ascidian *Eudistoma toealensis* and its predatory flatworm *Pseudoceros* sp. [26]. Furthermore, staurosporine and its derivatives have aroused considerable interest as these compounds exhibit strong inhibitory activities against protein kinase C as well as inhibition of platelet aggregation, blocking of growth phases in cancer cells and reversal of multidrug resistance [27]. In this study, stauroporine exhibited significant anti-parasitic activity against *Leishmania major* (IC_50_ 5.30 μM) and *Trypanosoma brucei brucei* (IC_50_ 0.022 μM) which has not been previously reported in literature.

The third compound, butenolide (Figure 2), was isolated from *Streptomyces* sp. strain T03 with a molecular formula of C_13_H_22_O_3_Na (*m/z* 249.1447 for [M + Na]^+^, calculated 249.1467) exhibiting identical spectral data with published literature [28] (see Supplementary Information). This lactone-containing metabolite has also been previously isolated from a marine sediment-derived *Streptomyces* sp. strain M027750 [28]. Butenolides are a family of α,β-unsaturated lactones commonly produced by fungi, bacteria and gorgonians (colonial soft corals). Their saturated analogs act as signaling substances in bacteria, enhance spore formation of *Streptomyces* sp. as well as induce metabolite formation [29]. In this study, butenolide was found to exhibit anti-*Trypanosoma* activity (IC_50_ 0.022 μM).

The compounds valinomycin and staurosporine were found to exhibit general cytotoxicity against 293T kidney epithelial cells (valinomycin IC_50_ 11.24 μM; staurosporine IC_50_ 1.30 μM) and J774.1 macrophages (valinomycin IC_50_ < 0.10 μM; staurosporine IC_50_ < 0.13 μM) while butenolide was not found to exhibit cytotoxicity against these cell lines [30]. Nevertheless, these compounds have been shown to exhibit significant anti-parasitic activities (Table 1) which has not been previously reported. Structure modification of these compounds with the aim to decrease cytotoxicity is therefore a worthwhile endeavour. These results highlight the potential of actinomycetes associated with marine sponges to produce bioactive compounds. Furthermore, the re-isolation of previously known compounds is still considered a worthwhile pursuit particularly for finding new pharmacological uses such as anti-infectives. The emergence of antibiotic resistance and the alarming death rate caused by infectious diseases necessitates the need for re-evaluating the current multitude of compounds that have been discovered over the past years.

## Figures and Tables

**Table 1:** Anti-parasitic activities of the compounds (IC_50_, μM).

Compound	*Leishmania major*	*Trypanosoma brucei brucei* (48 h)	*Trypanosoma brucei brucei* (72 h)
valinomycin	<0.11	0.0032	0.0036
staurosporine	5.30	0.022	0.035
butenolide	>100	31.77	33.08
